# Role of the nervous system in cancers: a review

**DOI:** 10.1038/s41420-021-00450-y

**Published:** 2021-04-12

**Authors:** Huan Wang, Qiming Zheng, Zeyi Lu, Liya Wang, Lifeng Ding, Liqun Xia, Hao Zhang, Mingchao Wang, Yicheng Chen, Gonghui Li

**Affiliations:** Department of Urology, Sir Run Run Shaw Hospital, Zhejiang University School of Medicine, Hangzhou, Zhejiang 310016 China

**Keywords:** Cancer microenvironment, Targeted therapies

## Abstract

Nerves are important pathological elements of the microenvironment of tumors, including those in pancreatic, colon and rectal, prostate, head and neck, and breast cancers. Recent studies have associated perineural invasion with tumor progression and poor outcomes. In turn, tumors drive the reprogramming of neurons to recruit new nerve fibers. Therefore, the crosstalk between nerves and tumors is the hot topic and trend in current cancer investigations. Herein, we reviewed recent studies presenting direct supporting evidences for a better understanding of nerve–tumor interactions.

## Facts

1. Nerves, as components of the tumor microenvironment, are associated with cancer outcomes.
2. Nerve transmitters and neurotrophic factors play an essential role in tumor progression.
3. Perineural invasion is a common characteristic of some tumors.

## Open questions

1. How do tumors and nerves have crosstalk?
2. What is the molecular mechanism underlying of perineural invasion?
3. How can we appropriately target the nerves to prevent the tumor progression?

## Introduction

The tumor microenvironment (TME) is closely related to tumor initiation, progression, and metastasis. It consists of the extracellular matrix, fibroblasts, adipose cells, immune-inflammatory cells, blood, and lymphatic vascular networks^1^. The functions of oncogenes and tumor suppressor genes in tumorigenesis have long been identified. In recent years, the concept of cancer biology has shifted from studying the genetics of tumor cells alone to the field of complicated interplay between tumor cells and the TME. The elements of this interplay, especially tumor angiogenesis, have been well-characterized in previous research^2^. Hence, nerves as components of the TME have been increasingly proved to regulate aberrant tissue function, including cancer progression. The crosstalk between nerves and cancer cells has been well-established for a variety of cancers, including pancreatic, prostate, breast, head and neck cancers, as well as cholangiocarcinoma^3–7^. This association is often correlated with poor outcomes. Upon the recognition of the significance of nerve–cancer interactions, the National Cancer Institute has convened their first meeting to explore the “Role of Nerves in Cancer progression” in March 2015^8^.

Many research groups have established various models and demonstrated their own hypothesis to answer the main relevant questions, such as the influence of neuroactive molecules on cancers, the contribution of different nerves to cancers, or how cancers and nerves communicated. Technological advances in precise nerve imaging and manipulation has also allowed some progress in understanding the molecular mechanisms behind the crosstalk between cancers and nerves.

In this review, current theories on the communication channels and the functional relationship between cancers and nerves are summarized. The cellular and molecular mechanisms of the process based on recent studies are also reviewed. Thus, a more comprehensive understanding of the interplay between tumor cells and nerves may be useful to devise new strategies for cancer therapy.

## Nerves create a unique TME and associate with the outcome

Nerves, consisting of a variety of cells such as neurons and neuroglia, form a unique type of TME. Nerves and related nerve cell markers have been detected in various tumors, including those of the head and neck, prostate, breast, cervix, esophagus, stomach, colorectum, and pancreas^9–15^. When cancers develop from preneoplastic lesions to obvious cancer, nerve density nearly doubles compared with the non-neoplastic tissue^16^. More importantly, nerves found in the tumor increase the malignancy and are often correlated with poor outcomes. Huang et al.^10^ confirmed that nerve fibers in breast cancer were significantly correlated with poor differentiation, lymph node metastasis, high clinical staging, and the triple-negative subtype. In the same manner, nerve fiber density was also correlated with tumor size, margin status, lymph node metastasis, pathological tumor, and American Joint Committee on Cancer stages, as well as survival time in pancreatic cancers^7,15^. A further study also found nerves to be involved in angiogenesis related to tumor growth^17^. Horn et al.^18^ associated nerves with the recurrence of rectal adenocarcinoma after the radical surgery. All the above evidences support that nerves play crucial roles in tumor progression.

Tumor–nerve interactions are characterized by two aspects as follows: (i) tumor cells can secrete neurotrophic factors, neurotransmitters, and axon guidance molecules via paracrine signaling to drive neuron reprogramming, and thereby recruit the nerves or invade the existing nerves; (ii) nerves can also secrete neuroactive molecules to interact with the receptors of tumors or the TME for the tumor cells to proliferate, invade, and metastasize. These two aspects will be discussed in detail below.

## Effect of tumor cells on nerves

### Perineural invasion in cancers

Cancer cells infiltrate inside or around nerves in the process of perineural invasion (PNI), which can be observed before lymphatic or vascular invasion^19,20^. The PNI process, first characterized in head and neck carcinoma in 1856 by Batsakis^19^, has been described in detail by several reviews^20–24^. The first definition, however, was unclear and PNI attracted little attention in subsequent research. In 2009, Liebig et al.^20^ summed up previous studies and characterized PNI by the close proximity of tumor to nerve, and as a process involving at least 33% of its circumference or tumor cells present within any of the three layers of the nerve sheath. Thus, PNI is more commonly detected in aggressive cancers. The incidence of PNI was reported as up to 80% in head and neck cancers, 75% in prostate cancers, 98% in pancreatic cancers, 33% in colorectal cancers^20^, and 75% in cholangiocarcinoma^25^. Evidences indicating that PNI is a significant predictor of overall survival or disease-free survival of tumor patients is emerging^9,26–30^.

**Fig. 1:**
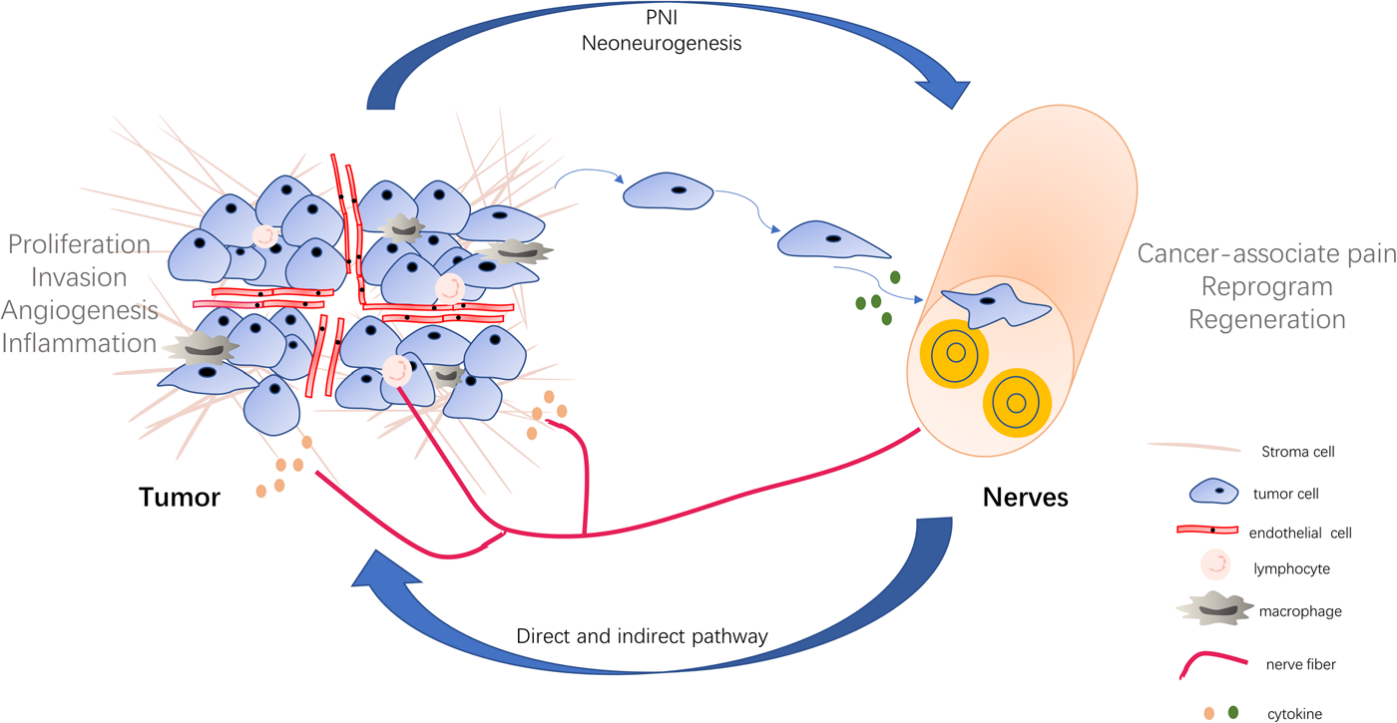
The nerve–tumor crosstalk. Nerves can secrete the neuroactive molecules that act on tumor cells, lymphocytes, and macrophages to promote tumor proliferation, invasion, angiogenesis, and inflammation. In turn, tumor cells migrate to nerves and damage them to induce the cancer-associated pain. Moreover, tumor cells secrete cytokines, which drive nerve reprogramming and regeneration. PNI perineural invasion.

PNI involving structural nerve damage directly leads to cancer-associated pain^21^. More importantly, it is considered a potential pathway for cancer cells dissemination and metastasis in the same way as vascular and lymphatic channels^31^. Saloman et al.^3^ showed that tumor cells invaded neural tissue before the onset of tumorigenesis. In addition, at the early stage of cancer, pancreas acinar-derived cells were found to migrate along sensory neurons into the spinal cord, providing evidence that PNI could be a potential route of metastasis^3^. Recent studies suggest that the neural tracking hypothesis likely explains the mechanisms of PNI. Cancer cells track along or around a nerve after infiltrating into perineural space in the process of nerve injury, which in turn promotes neural regeneration. The damage of perineurium caused by invading cancer cells leads to a cascade of inflammation cytokines, thus forming a unique cellular and biochemical microenvironment around the nerve, named perineural niche^32–35^. This perineural niche includes various cellular components and may regulate the neural tracking to facilitate the PNI^36,37^ (Fig. 1).

In summary, PNI is a special means of tumor–nerve communication that is correlated to tumor progression. However, the understanding of PNI mechanisms remains limited, as an appropriate model to imitate the complex interactions between tumor and nerve fibers has not been proposed.

### Neoneurogenesis

Neoneurogenesis (also called innervation) is the process when cancer cells react to nerve regeneration and recruit new axons into the tumor tissue, similar to angiogenesis. In contrast to the central nervous system cells, peripheral nerve cells have the ability to regenerate after injury^38^. Neoneurogenesis is a highly complex biological phenomenon, which remains to be elucidated. The neo-nerves originating from different nerves play different roles, or even opposite roles, in various tumors; the corresponding results are presented in Table 1.

**Table 1:** Neurogenesis in various cancers and potential mechanisms.

Cancer type	Associated tumor nerves	Outcomes	Mechanisms	Reference
Prostate cancer	Sympathetic nerves Parasympathetic nerves	Promote tumorigenesis and dissemination Indicate poor clinical prognosis	Sympathetic nerve functions through Adrb2 and Adrb3 receptors. Parasympathetic nerve functions through Chrm1 receptor.	^6^
Basel cell carcinoma	Sensory nerves	Promote tumor formation	Sensory nerves activate Hedgehog signaling in normal touch domes.	^48^
Gastric cancer	Vagus nerves	Promote gastric tumorigenesis Influence the therapeutic effects of systemic chemotherapy	Active Wnt signaling through cholinergic receptor. Stimulate the YAP function.	^50,52^
Pancreatic ductal adenocarcinoma (PDAC)	Sensory nerves Vagus nerves	Sensory nerves promote initiation and progression of the early stages of PDAC. Vagus nerves inhibit the tumor growth and prolong survival time.	Sensory neurons convey inflammatory signals to drive inflammation. Vagus nerves regulate tumor-associated macrophages and TNFα signaling.	^3,47,53^
Breast cancer	Sympathetic nerves Parasympathetic nerves	Sympathetic nerves accelerate breast cancer growth and progression. Parasympathetic nerves reduced breast cancer growth and progression. The thickness of tumor-involving nerve fibers is correlated with lymph node metastasis, clinical stage, and survival time.	Regulate the expression of immune checkpoint molecules PD-1, PD-L1, and FOXP3.	^4,10^
Head and neck cancer	Sensory nerves	Promote tumor progression	Sensory nerves differentiate into adrenergic neo-neurons induced by the tumor to promote tumor progression.	^5^
Glioma	Central nervous system neuron	Promote glioma progression	Neurons promote tumor progression through AMPA receptor-dependent neuron-glioma synapses.	^76,77^

The peripheral nerve system (PNS) includes the sympathetic and parasympathetic nerves, and maintains the homeostases of the body. The neurotransmitter of the sympathetic nerves is norepinephrine, whereas that of the parasympathetic nerves is acetylcholine (Ach), which both play important roles in the cellular communication. These coordinated systems control the blood pressure, pH, thermoregulation, and metabolism, to adapt to external and internal pressures^39^. Sympathetic and parasympathetic nerves usually have opposing effects on a given tissue, such that they increase the activity of one system, whereas decrease the activity of the other. More specifically, sympathetic nervous activity increases the flow of blood rich in nutrients to tissues that need it during emergency “fight-or-flight” reactions. Meanwhile, the parasympathetic system predominates during quiet, resting conditions. In summary, the PNS is affected by the tissues it innervates and responds to the changes in the microenvironment^40^. Therefore, certain lines of evidences have linked neoneurogenesis in a tumor to the PNS, including both sympathetic and parasympathetic nerves^41^. Prostate cancer neoneurogenesis was recognized relatively early^42^, whereas it was only later that Magnon et al.^6^ first demonstrated that the formation of autonomic nerve fibers in prostate cancer was required for cancer development and progression. In various mouse models, sympathetic nerve ablation, including that by both chemical or surgical method, could prevent prostate cancer development. Also, parasympathetic destruction could suppress the dissemination and invasion of prostate cancer. In a retrospective study, a high density of sympathetic and parasympathetic nerves was detected in tumors, and this was associated with poor clinical outcomes. Consistently with the results for prostate cancer, sympathectomy by the bilateral removal of superior cervical ganglia inhibited the tongue tumor growth and invasiveness^43^. Interestingly, breast cancer growth and progression were accelerated after sympathetic nerves stimulation, but were suppressed following the stimulation of parasympathetic nerves^4,10,44^. These findings suggested that the innervation of sympathetic and parasympathetic nerves play different roles in different cancers.

In addition to the parasympathetic and sympathetic nerves, sensory nerves also participated in the tumor progression. For example, sensory nerves may drive inflammation to accelerate precancerous lesions to pancreatic cancer via neurogenic mechanisms^45,46^. At the early stage of pancreatic cancer, the expressions of pancreatic neurotrophic factors change and sensory innervation obviously increases. At later stages, cells of pancreatic origin could migrate to the sensory ganglia and the spinal cord. The above findings prove that the sensory nerves participated in all stages of pancreatic cancers including tumorigenesis and progression^47^. Furthermore, the ablation of sensory neurons in a genetic model of pancreatic cancers showed a suppressive effect on tumor initiation and progression^3^. In a similar manner, sensory neurons were also shown to play a direct role in tumor formation in basal cell carcinoma^48^.

The vagus nerve, which contains both parasympathetic and sensory axons in a mixed nerve^49^, has been demonstrated to play completely inverse roles in pancreatic ductal adenocarcinoma and gastric cancer. Using three models of gastric cancer, Zhao et al.^50^ proved that vagotomy or pharmacological denervation of only the stomach portion decreased tumor progression and prolonged survival when performed in later cancer stages. Denervation particularly affected the renewal of the stem cell compartment of gastric tumors and was also able to enhance the effect of chemotherapy^50^. In addition to the vagus nerve, enteric nerves are also involved in gastric cancer initiation and progression^51,52^. In contrast to the cancer-inducing effects in the gastric cancer, vagus nerves had an antitumor effect in pancreatic cancer^53,54^. Vagotomy accelerated pancreatic tumorigenesis and enhanced tumor growth through recruiting tumor-associated macrophages (TAMs) and mediating the inflammation^55,56^.

According to novel findings, newly formed adrenergic nerve fibers in neck and head cancer originated from the sensory neurons and are not the infiltrations of pre-existing adrenergic nerves^5^. Hence, signals that promoted tumor growth are also regulated by newly formed adrenergic nerve fiber instead of pre-existing adrenergic nerves. Furthermore, Amit et al.^5^ identified the adrenergic differentiation signature by comparing the transcriptomes of cancer-associated neurons with those of endogenous neurons. Their investigation of mechanisms revealed that *TP53* loss in head and neck cancer drives sensory nerves reprogramming through the delivery of cancer-derived exosomes lacking miR-34a. Moreover, tumor growth was inhibited by sensory denervation or the pharmacological blockade of adrenergic receptors, but not by the chemical sympathectomy of pre-existing adrenergic nerves. These results indicated that cancer cells drive the neuron reprogramming to promote tumor progression. However, the potential role of neuron reprogramming induced by cancer cells in other tumors remains to be established.

Another study revealed that nerves emerging in tumors can also originate from the central nervous system^57^. In mouse models of prostate cancer, neural progenitors expressing doublecortin (DCX+) in the subventricular zone, egress into the circulation through disrupting the blood–brain barrier. These cells then infiltrate and reside in the tumor and can generate new neurons. Hence, the genetic depletion of DCX+ cells inhibit the prostate cancer progression, whereas DCX+ cells transplantation promotes prostate tumor growth and metastasis. These results provide new insights into the origin of tumor nerves, but the mechanisms of how solid tumors communicate with the central nervous system remain to be elucidated. In addition, the seeds of neoneurogenesis in tumors can also be mesenchymal stem cells (MSCs) derived from the bone marrow. Tumors recruited MSC, which in turn can differentiate into neurons under proper conditions in the TME^58^.

In summary, PNI and neoneurogenesis often occur and function together, thus providing the structural foundation for tumor–nerve communication.

## Direct effects of nerves on tumor

Tumors affect nerve behavior but, more importantly, nerves also play important roles in regulating tumor progression through direct or indirect pathways. As cell membranes in tumors bear receptors that respond to neurotropic factors or neurotransmitters, the nerves release such molecules to promote tumor progression in a paracrine manner. Hence, innervations can release neurotransmitters directly into a synapse formed by neurons and tumor cells to transfer the excitatory signal. In the following section, we describe the two main direct effects of nerves on tumor progression.

### Paracrine mode

**Fig. 2:**
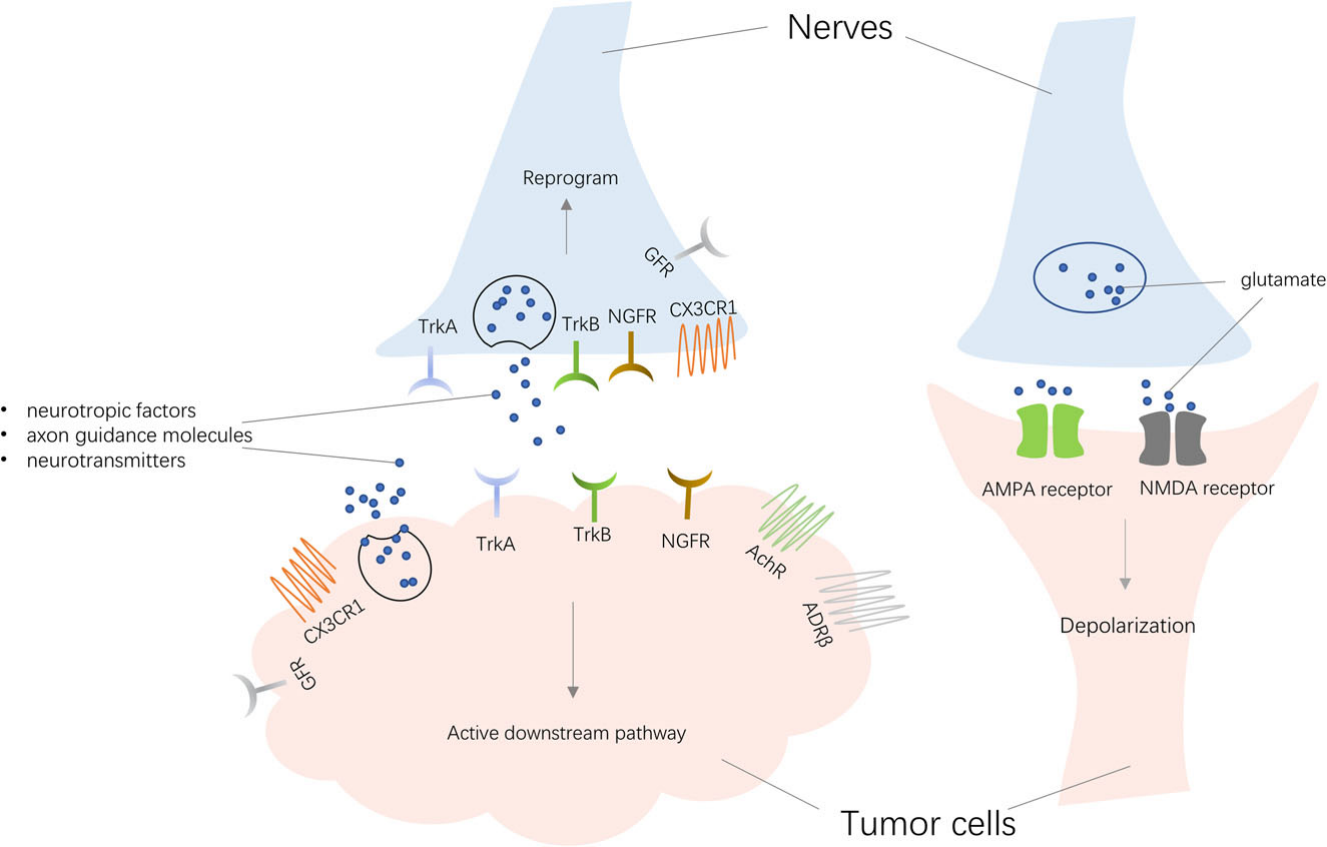
Two direct ways of communication between tumor cells and nerves. Tumors release molecules also secreted by nerves, including neurotropic factors, axon guidance molecules, and neurotransmitters. These factors act on receptors of nerves to drive the nerve reprogramming, while they can also act on tumor receptors to activate downstream signaling. In addition to the paracrine model, tumors and nerves can form the synapses for depolarization to promote tumor progression. TrkA tyrosine kinase receptor A, TrkB tyrosine kinase receptor B, NGFR nerve growth factor receptor, AchR acetylcholine receptor, ADRβ adrenoceptor β receptor, CX3CR1 C-X3-C motif chemokine receptor 1, GFR glial cell-derived neurotrophic factor family receptor.

Nerves, including neurons and Schwann cells, can modulate the biological behavior of cancer cells and influence tumor progression through the paracrine mode. From a broad perspective, neuroactive molecules released by the nerves involved in tumor–nerve interaction can be divided into three main families as follows: (i) neurotropic factors, such as nerve growth factor (NGF), brain-derived neurotrophic factor (BDNF), glial cell line-derived neurotrophic factor, and others; (ii) axon guidance molecules, such as CCL2, CX3CL1, EphA2, Slit, etc.; and (iii) neurotransmitters, including Ach, glutamate, glycine, epinephrine, norepinephrine, dopamine, etc.^8,59^. Unsurprisingly, tumor cells express various receptors, such as tyrosine kinase receptor A (TrkA), TrkB, and NGF receptor (NGFR), when responding to different neuroactive molecules to activate downstream pathways. Decades of research revealed the neuroactive molecules and receptors associated with tumor progression^22,24,60–63^. This way of communication between tumor cells and nerves is shown in Fig. 2.

In the prostate cancer model^6^, adrenergic fibers play a significant role through β2- and β3-adrenergic receptors, whereas the cholinergic fibers act through the cholinergic receptors. The fact that β-blocker use can prolong survival in high-risk or metastatic prostate cancer patients is consistent with these findings^64^. Similar effects were also observed in skin cancer and breast cancer patients taking adrenergic antagonists^65–67^. Subsequently, Hayakawa et al.^52^ discovered that cholinergic stimulation by nerves through the release of Ach induced NGF expression in the gastric epithelium. In turn, NGF overexpression promoted cancer progression^52^. Using the The Cancer Genome Atlas data, Deborde et al.^68^ revealed that a total of 48% of pancreatic cancer patients showed an alteration in genes coding neuroactive molecules or their receptors. The alternation of these genes was similar at 48% of patients with neuroendocrine prostate cancer and at 67% of patients with breast cancer. The gene whose expression changed most obviously was *NTRK1* coding TrkA^24^. A further study demonstrated that TrkA expression could be found in 1.6% of solid tumors and was paralleled by the number of *NTRK1* gene copies^69^. Inhibitors of TrkA have already shown a potential to treat *NTRK* fusion-positive cancer^70^.

The neurotransmitter ACh acts as an autocrine growth factor in human lung cancer and pancreatic cancer. Song et al. showed that ACh stimulated the proliferation of lung cancer cells via activating the mitogen-activated protein kinase and AKT pathways^71,72^. Apart from proliferation, ACh potently stimulates the adhesion, migration, and invasion of human lung cancer cells. Lin et al.^73^ observed that ACh increased the expression of MMP9 and downregulated the expression of E-cadherin. Both of these signaling events were associated with the migration and invasion phenotype in lung cancer. In another study, the stimulation of 7-nAChR enhanced pancreatic cancer metastasis via activating the JAK2/STAT3 signaling cascade and the Ras/Raf/MEK/ERK1/2 pathway^74^.

The above findings revealed that neuroactive molecules and receptors both participate in the tumor progression through the paracrine mode.

### Chemical synapse

A special form of crosstalk between tumors and nerves is the chemical synapse, which is a structure that usually involves two adjacent neurons communicating using neurotransmitters, such as glutamate^75^. Synaptic structures involving presynaptic neurons and postsynaptic tumor cells in glioma were also observed by electron microscopy^76,77^. More importantly, by recording the excitatory postsynaptic potentials in glioma cells, both Venkatesh et al.^76^ and Venkataramani et al.^77^ indicated that these neurogliomal synapses may be functional in a similar manner to those formed between neurons. Gene expression analysis and confocal microscopy revealed that AMPA receptor was common in the postsynaptic region of glioma cells. Further studies on this receptor indicated that it mediated the depolarization of a glioma cell, which then spread through the network of glioma cells through their gap junctions. Crucially, neuronal activity or depolarization could promote tumor proliferation and invasion while preventing depolarization induced by synaptic activity, thus leading to a smaller tumor burden and longer survival time of animals^78–80^.

Zeng et al.^81^ also revealed that breast-to-brain metastasis (B2BM) cells establish pseudo-tripartite synapses between two neurons through the expression of neuroligin, which aids cell adhesion similar to the glioma^81–83^. High levels of the NMDA receptor (NMDAR)—in particular the subunit GLuN2B—was identified in B2BM cells through transcriptomic data. Furthermore, the currents and calcium transients after NMDAR activation were recorded. Cells produced smaller brain tumors and the mice had longer survival times after knocking down GLuN2B, suggesting that NMDAR synapses may promote the growth of cancer cells in the brain.

Taken together, these results demonstrate that nerves communicate with the tumors through establishing functional synapses to boost tumor progression. However, these tumors all occur in the brain environment and it remains unknown whether other solid tumors form synapses with nerves.

## Indirect effects of the nerves on tumor

**Fig. 3:**
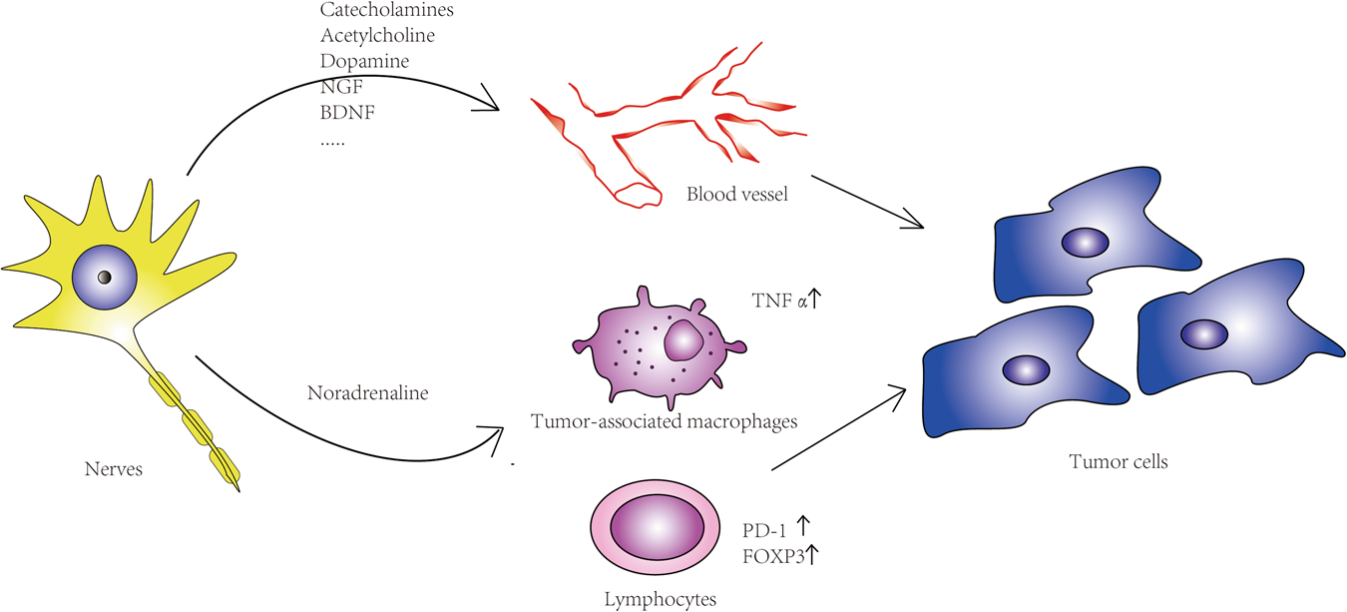
Nerves affect tumor cell behaviors through indirect pathways. Nerves induce angiogenesis through secreting transmitters and neurotrophic factors. On the other hand, they regulate tumor-associated macrophages via cholinergic and adrenergic signaling. In addition, nerves mediate the expression of immune checkpoint molecules (PD-1, PD-L1 FOXP3) by lymphocytes; all the above processes influence tumor behaviors.

Nerves also interact with multiple stromal components in the TME to indirectly promote tumor growth and metastasis. Previous studies have proved that nerves directly regulate stromal structures^84^. In this section, interactions between nerves and stromal compartments are discussed (Fig. 3).

### Angiogenesis

The process of angiogenesis, which is the growth of new capillary vessels from existing vasculature by the activation, proliferation, and migration of endothelial cells, plays a crucial role in tumor growth and metastasis^85–88^. It allows tumors to develop their own nutrients and oxygen supply, thus enabling cell proliferation and tumor growth. Angiogenesis reflects the aggressiveness of tumor cells and associated with tumor outcomes^89,90^.

Transmitters and neurotrophic factors secreted by the nerves are involved in the process of angiogenesis through binding to receptors and inducing endothelial cells migration^59^. These factors, including catecholamines, Ach, dopamine, NGF, BNDF, etc., have been well summarized by Kuol and colleagues^59,91–95^. Recently, Zahalka et al.^17^ revealed that adrenergic nerves regulated angiogenesis in the prostate cancer microenvironment by altering the metabolism of blood vessel endothelial cells. Mechanistically, ADRB2 inhibited endothelial oxidative phosphorylation, which led to angiogenesis. The metabolic shift induced by nerves promoted prostate tumor growth through angiogenesis^17^.

Angiogenesis and neoneurogenesis indeed share a number of similarities. Both processes are regulated by similar transmitters and neurotrophic factors, and even share the same receptors^96^. All the above findings demonstrate that the regulation of angiogenesis and neoneurogenesis are closely intertwined.

### Immunity

**Fig. 4:**
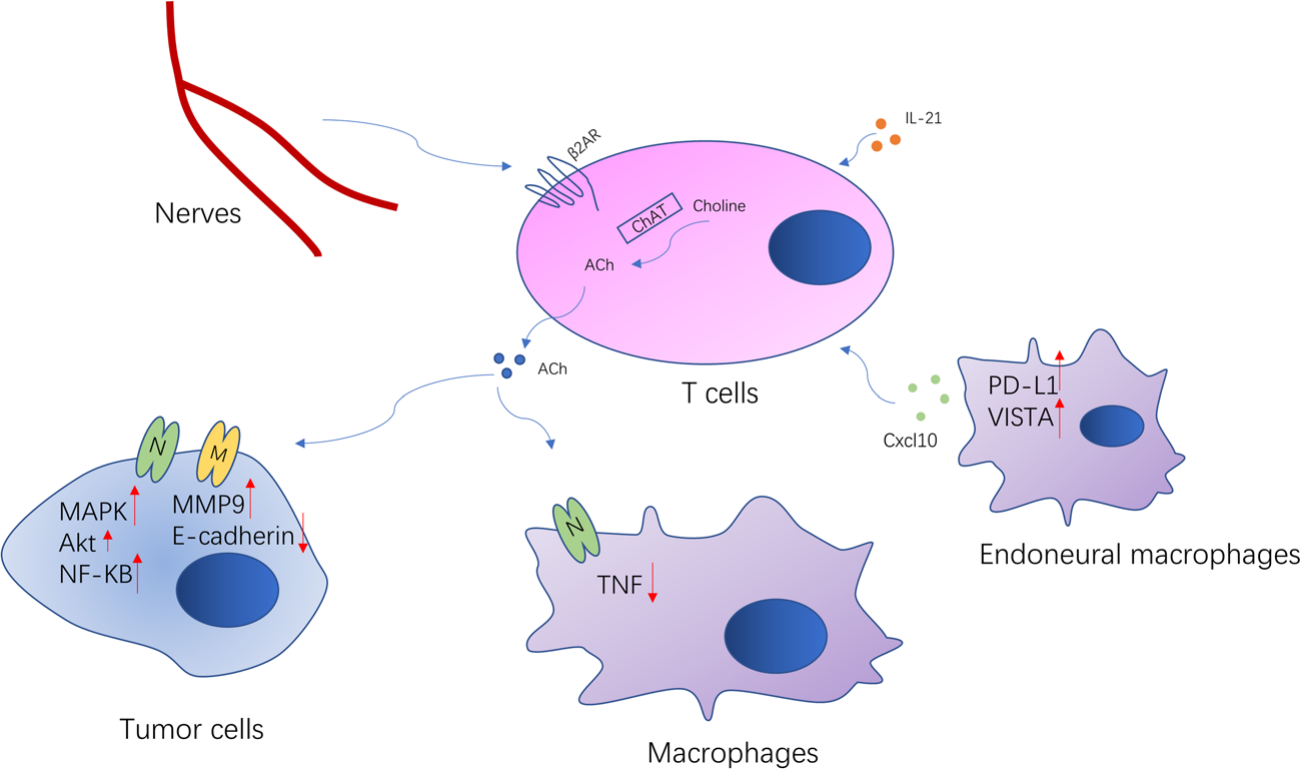
Crosstalk between nerves and immune cells. ACh is synthesized in the T cells by enzyme choline acetyltransferase. Adrenergic innervation stimulates production of the T-cell-derived ACh, which is involved in regulating the tumor immunity and tumor biology behavior. Endoneural macrophages also regulate tumor metastasis through CXCL10. CXCL10 recruits the myeloid cells and suppresses T cells via VISTA and PD-L1.

Nerves, as components of the TME, are also in crosstalk with the immune system, which could contribute to the tumor progression via inflammation^97^. These complex systems interact at multiple levels. Neuroendocrine and neuronal pathways are involved in the control of immune responses, as most of their molecular signals and receptors come from the same superfamily. In the spleen, e.g., adrenergic innervation was found to stimulate ACh production in β2-adrenergic receptor (β2-AR)-expressing T cells^98^. T-cell-derived ACh has been recently reported to play an important role in regulating immunity, including cancer immunity. T-cell-derived ACh can inhibit tumor necrosis factor (TNF) production through the α7 nicotinic Ach receptor expressed by cytokine-producing macrophages^99^. The released ACh also binds back to nicotinic and muscarinic receptors on lung cancer cells to accelerate their proliferation, migration, and invasion^100^. Choline acetyltransferase, which catalyzes the synthesis of ACh from choline, is indeed strongly induced in both CD4+ and CD8+ T cells via IL-21, to regulate T-cell migration and immune functions^101^ (Fig. 4). In conclusion, these studies showed that the autonomic nervous system can directly regulate the immune system.

Tumor lymphocyte infiltration and activation are the important processes to inhibit tumor growth and progression^102^. However, tumor cells escape immunosurveillance through the activation of immune checkpoint pathways that suppress antitumor immune responses^103,104^. A retrospective analysis of breast cancer patient revealed that sympathetic and parasympathetic nerve density correlated with the expression of immune checkpoint molecules (PD-1, PD-L1, and FOXP3) and clinical outcomes^4^. This effect was also observed in animal experiments. Genetic sympathetic nerve denervation and parasympathetic neurostimulation reduced the expression of immune checkpoint molecules in a tumor tissue-specific and fiber type-specific manner in animal breast cancer models. These findings partly explained the opposing effects of sympathetic and parasympathetic nerves in breast cancer, and indicated that nerves have a close association with immune checkpoint therapy. Nevertheless, the mechanism of how nerves regulate immune checkpoint molecules requires further elucidation.

TAMs, as essential components of the cancer microenvironment, play critical roles in the regulation of tumor development and progression^105^. TAMs can also modify the ability of tumor cells to resist cytotoxic chemotherapy via mediating the TME^106^. Interestingly, TAM recruitment is also regulated by both cholinergic and adrenergic signaling, which are related to the nerves. In pancreatic cancer, adrenergic signaling promotes tumor growth and reduces survival via TAM recruitment, while cholinergic signaling has the opposite effects^54,107^. A further study revealed that vagotomy promoted pancreatic cancer growth and reduced survival time through mediating TNFα secterion by TAMs^53^. Similar results were observed in the breast cancer^108^. Stress-induced neuroendocrine activation induced breast cancer metastasis to distant tissues, including the lung and lymph nodes. The pharmacological activation of β-adrenergic signaling induced similar effects, whereas a β-antagonist reversed these effects. Specifically, adrenergic signaling increased the infiltration of CD11b+ F4/80+ macrophages into the primary tumor and thereby induced a metastatic gene expression signature, accompanied by M2 macrophage differentiation.

Endoneural macrophages also participate in tumor metastasis. Microglia, as a type of native macrophages of the nervous system, are key promoters of brain metastasis^109^. Guldner et al. demonstrated that endoneural macrophages in the central nervous system drive immune suppression in the brain metastases through CXCL10. Furthermore, macrophages could suppress T-cell activation to promote the brain metastases via VISTA and PD-L1^110^. The elimination or inhibition of microglia function resulted in good antitumor metastasis effect. The blocking of any of the CCL2, STAT3, CSF-1R, and PI3K pathways of macrophages could inhibit brain metastasis^111–114^. However, more detailed investigations are needed to clarify the role of endoneural macrophages in tumorigenesis.

In summary, results suggest that nerves can regulate tumor progression through affecting the immune cells.

## Neural regulation in treatment resistance

Owing to the deeper understanding of the underlying biological processes and molecular mechanisms of cancer progression, great progress has been made in cancer treatment. However, sooner or later, resistance develops to all kinds of therapy. Recent studies suggest that nerves and neural signals manipulate cancer therapeutic resistance^115,116^. This section aims to discuss the association between neural regulation and treatment resistance.

Chemotherapy is one of the most important applied therapeutic strategies for most cancers. Response to chemotherapeutics, however, varies greatly between individuals. Accumulating evidences suggest that the aberrant activation of adrenergic signaling affect sensitivity to cytotoxic chemotherapeutics by modulating the expression of other anti-apoptotic genes and inhibiting cellular apoptosis. Eng et al.^117^ reported that the activation of β2-ARs resulted in changes in the apoptotic pathway regulation, which led to reduced therapeutic response. In cervical cancer cells, β2-AR activation also induced chemoresistance by modulating p53 acetylation through upregulating SIRT1^118^. A further study also indicated that β2-AR remarkably impaired the chemotherapy response via upregulating DUSP1^119^. These findings suggest that poor response to chemotherapeutics may be partly attributed to the abnormal functional activities of adrenergic signals.

Growing evidence reveals that nerves also stimulate a wide variety of signaling pathways causing resistance to drug therapy. Targeting members of the Epidermal Growth Factor Receptor family is an effective strategy for treating various cancers. Trastuzumab is the first line of therapy for Her2-positive breast cancer and gastric cancer^120,121^. However, Trastuzumab resistance is a major clinical problem in the treatment of cancers^122^. Further evidence has suggested that β2-AR is involved in the mechanism of Trastuzumab resistance. Shi et al. revealed that β2-AR expression was positively correlated with Her2 expression in breast cancer; β2-AR and Her2 comprised a positive feedback loop, where Her2 induced the upregulation of β2-AR via ERK pathway, whereas β2-AR induced the upregulation of Her2^123^. Moreover, β2-AR resulted in Trastuzumab resistance through mediating the PI3K/AKT/mTOR pathway. Retrospective studies have also demonstrated that combining β-blockers with trastuzumab significantly improved survival in the patients with metastatic breast cancer^124^. It has also been reported that the activation of the β2-AR signaling confers resistance to the tyrosine kinase inhibitor in the non-small cell lung cancer and hepatocellular carcinoma^125,126^.

Taken together, these results revealed that neural regulation was involved in tumor treatment resistance and targeting neural signaling pathway might be a potential strategy for treatment resistance.

## Conclusions

In this review, it was highlighted that tumors can affect the nerves, which in turn, may modulate tumor biology via direct or indirect pathways. The detailed mode of tumor–nerve interaction was presented in Fig. 1. All of the listed evidences indicate that the nervous system is not a bystander with regards to cancer development and progression. Furthermore, some lines of evidences have linked the nerves to the treatment resistance. Due to the intimate relationship between nerves and tumor behavior, targeting nerves may provide novel strategies for the treatment of highly innervated cancers. As a result of nervous regulation of tumor angiogenesis and immunity, nerve targeting strategies could also be combined with anti-vascular therapy or immune therapy for a better cancer treatment effect. In recent years, the nerve targeting approach has already been applied in some clinical trials, but relevant methods are still far from clinical application.

For instance, surgical denervation significantly reduced gastric tumor incidence and progression^50^. However, the assessment of benefits and side effects of the procedure needs further investigation. The pharmacological inhibition of neural signaling is a promising target in anti-cancer therapy. Adrenergic signaling plays a critical role in tumor progression; thus, cancer treatment using β-adrenergic blockers remains controversial. Several lines of evidence have suggested that β-adrenergic blockers could prevent or reduce the mortality of various cancers, such as those of the pancreas, breast, and prostate^127,128^. Meanwhile, Heitz et al. claimed that selective β-blockers intake did not influence the prognosis for ovarian cancer patients.^129^ Regarding neurotrophic factors, NGF, BDNF, and their TrK receptors are the current research hotspots. However, the small-molecular targeting of TrKs has not been shown to have an impact on patient survival in clinical trials^130,131^; they even exhibit certain side effects by affecting other tyrosine kinases^132^. As a whole, further research is needed to identify the mechanisms of targeting the nerve pathway more specifically and to identify cancer patients that would benefit denervation procedures.

## References

[1] Chen F (2015). New horizons in tumor microenvironment biology: challenges and opportunities. BMC Med..

[2] Wang Y, Wang L, Chen C, Chu X (2018). New insights into the regulatory role of microRNA in tumor angiogenesis and clinical implications. Mol. Cancer.

[3] Saloman JL (2016). Ablation of sensory neurons in a genetic model of pancreatic ductal adenocarcinoma slows initiation and progression of cancer. Proc. Natl Acad. Sci. USA.

[4] Kamiya A (2019). Genetic manipulation of autonomic nerve fiber innervation and activity and its effect on breast cancer progression. Nat. Neurosci..

[5] Amit M (2020). Loss of p53 drives neuron reprogramming in head and neck cancer. Nature.

[6] Magnon C (2013). Autonomic nerve development contributes to prostate cancer progression. Science.

[7] Tan X (2020). Nerve fibers in the tumor microenvironment in neurotropic cancer-pancreatic cancer and cholangiocarcinoma. Oncogene.

[8] Saloman JL, Albers KM, Rhim AD, Davis BM (2016). Can stopping nerves, stop cancer?. Trends Neurosci..

[9] Liebig C (2009). Perineural invasion is an independent predictor of outcome in colorectal cancer. J. Clin. Oncol..

[10] Huang D (2014). Nerve fibers in breast cancer tissues indicate aggressive tumor progression. Medicine.

[11] Hibi T (2009). Synuclein-gamma is closely involved in perineural invasion and distant metastasis in mouse models and is a novel prognostic factor in pancreatic cancer. Clin. Cancer Res..

[12] Horn LC, Meinel A, Fischer U, Bilek K, Hentschel B (2010). Perineural invasion in carcinoma of the cervix uteri-prognostic impact. J. Cancer Res. Clin. Oncol..

[13] Tianhang L, Guoen F, Jianwei B, Liye M (2008). The effect of perineural invasion on overall survival in patients with gastric carcinoma. J. Gastrointest. Surg..

[14] Chen JW (2014). The prognostic effect of perineural invasion in esophageal squamous cell carcinoma. BMC Cancer.

[15] Chatterjee D (2012). Perineural and intraneural invasion in posttherapy pancreaticoduodenectomy specimens predicts poor prognosis in patients with pancreatic ductal adenocarcinoma. Am. J. Surg. Pathol..

[16] Ayala GE (2008). Cancer-related axonogenesis and neurogenesis in prostate cancer. Clin. Cancer Res..

[17] Zahalka AH (2017). Adrenergic nerves activate an angio-metabolic switch in prostate cancer. Science.

[18] Horn A, Dahl O, Morild I (1991). Venous and neural invasion as predictors of recurrence in rectal adenocarcinoma. Dis. Colon Rectum.

[19] Batsakis JG (1985). Nerves and neurotropic carcinomas. Ann. Otol. Rhinol. Laryngol..

[20] Liebig C, Ayala G, Wilks JA, Berger DH, Albo D (2009). Perineural invasion in cancer: a review of the literature. Cancer.

[21] Bapat AA, Hostetter G, Von Hoff DD, Han H (2011). Perineural invasion and associated pain in pancreatic cancer. Nat. Rev. Cancer.

[22] Arese M, Bussolino F, Pergolizzi M, Bizzozero L, Pascal D (2018). Tumor progression: the neuronal input. Ann. Transl. Med..

[23] Demir IE (2010). Neural invasion in pancreatic cancer: the past, present and future. Cancers.

[24] Deborde S, Wong RJ (2017). How Schwann cells facilitate cancer progression in nerves. Cell. Mol. life Sci..

[25] Mavros MN, Economopoulos KP, Alexiou VG, Pawlik TM (2014). Treatment and prognosis for patients with intrahepatic cholangiocarcinoma: systematic review and meta-analysis. JAMA Surg..

[26] Hirai I (2002). Perineural invasion in pancreatic cancer. Pancreas.

[27] Duraker N, Sişman S, Can G (2003). The significance of perineural invasion as a prognostic factor in patients with gastric carcinoma. Surg. today.

[28] He P (2002). Multivariate statistical analysis of clinicopathologic factors influencing survival of patients with bile duct carcinoma. World J. Gastroenterol..

[29] Lee IH (2007). Perineural invasion is a marker for pathologically advanced disease in localized prostate cancer. Int. J. Radiat. Oncol. Biol. Phys..

[30] Schmitd LB, Scanlon CS, D’Silva NJ (2018). Perineural invasion in head and neck cancer. J. Dent. Res..

[31] Amit M, Na’ara S, Gil Z (2016). Mechanisms of cancer dissemination along nerves. Nat. Rev. Cancer.

[32] De Oliveira T (2012). Syndecan-2 promotes perineural invasion and cooperates with K-ras to induce an invasive pancreatic cancer cell phenotype. Mol. Cancer.

[33] Marchesi F (2008). The chemokine receptor CX3CR1 is involved in the neural tropism and malignant behavior of pancreatic ductal adenocarcinoma. Cancer Res..

[34] Abiatari I (2009). Consensus transcriptome signature of perineural invasion in pancreatic carcinoma. Mol. Cancer Ther..

[35] Li X (2014). Sonic hedgehog paracrine signaling activates stromal cells to promote perineural invasion in pancreatic cancer. Clin. Cancer Res..

[36] Demir IE (2013). Perineural mast cells are specifically enriched in pancreatic neuritis and neuropathic pain in pancreatic cancer and chronic pancreatitis. PLoS ONE.

[37] Cavel O (2012). Endoneurial macrophages induce perineural invasion of pancreatic cancer cells by secretion of GDNF and activation of RET tyrosine kinase receptor. Cancer Res..

[38] Makwana M, Raivich G (2005). Molecular mechanisms in successful peripheral regeneration. FEBS J..

[39] Idiaquez J., Benarroch E. & Nogues M. In *Evaluation and Management of Autonomic Disorders: A Case-Based Practical Guide* (eds. Idiaquez J., Benarroch E. & Nogues M.) 3–18 (Springer, 2018).

[40] McCorry LK (2007). Physiology of the autonomic nervous system. Am. J. Pharm. Educ..

[41] McVary KT (1994). Growth of the rat prostate gland is facilitated by the autonomic nervous system. Biol. Reprod..

[42] McVary KT, McKenna KE, Lee C (1998). Prostate innervation. Prostate Suppl..

[43] Raju B, Haug SR, Ibrahim SO, Heyeraas KJ (2007). Sympathectomy decreases size and invasiveness of tongue cancer in rats. Neuroscience.

[44] Kappos EA (2018). Denervation leads to volume regression in breast cancer. J. Plast. Reconstruct. Aesth. Surg..

[45] Schwartz ES (2011). Synergistic role of TRPV1 and TRPA1 in pancreatic pain and inflammation. Gastroenterology.

[46] Schwartz ES (2013). TRPA1 and TRPA1 antagonists prevent the transition of acute to chronic inflammation and pain in chronic pancreatitis. J. Neurosci..

[47] Stopczynski RE (2014). Neuroplastic changes occur early in the development of pancreatic ductal adenocarcinoma. Cancer Res..

[48] Peterson SC (2015). Basal cell carcinoma preferentially arises from stem cells within hair follicle and mechanosensory niches. Cell Stem Cell.

[49] Berthoud HR, Neuhuber WL (2000). Functional and chemical anatomy of the afferent vagal system. Auton. Neurosci..

[50] Zhao CM (2014). Denervation suppresses gastric tumorigenesis. Sci. Transl. Med..

[51] Polli-Lopes AC, Zucoloto S, de Queirós Cunha F, da Silva Figueiredo LA, Garcia SB (2003). Myenteric denervation reduces the incidence of gastric tumors in rats. Cancer Lett..

[52] Hayakawa Y (2017). Nerve growth factor promotes gastric tumorigenesis through aberrant cholinergic signaling. Cancer Cell.

[53] Partecke LI (2017). Subdiaphragmatic vagotomy promotes tumor growth and reduces survival via TNFalpha in a murine pancreatic cancer model. Oncotarget.

[54] Renz BW (2018). Cholinergic signaling via muscarinic receptors directly and indirectly suppresses pancreatic tumorigenesis and cancer stemness. Cancer Discov..

[55] Zhu Y (2017). Tissue-resident macrophages in pancreatic ductal adenocarcinoma originate from embryonic hematopoiesis and promote tumor progression. Immunity.

[56] Partecke LI (2017). Subdiaphragmatic vagotomy promotes tumor growth and reduces survival via TNFα in a murine pancreatic cancer model. Oncotarget.

[57] Mauffrey P (2019). Progenitors from the central nervous system drive neurogenesis in cancer. Nature.

[58] Scuteri A (2011). Mesenchymal stem cells neuronal differentiation ability: a real perspective for nervous system repair?. Curr. Stem Cell Res. Ther..

[59] Kuol N, Stojanovska L, Apostolopoulos V, Nurgali K (2018). Role of the nervous system in tumor angiogenesis. Cancer Microenviron..

[60] Mancino M, Ametller E, Gascón P, Almendro V (2011). The neuronal influence on tumor progression. Biochim. Biophys. Acta.

[61] Barquilla A, Pasquale EB (2015). Eph receptors and ephrins: therapeutic opportunities. Annu. Rev. Pharmacol. Toxicol..

[62] Rehman M, Tamagnone L (2013). Semaphorins in cancer: biological mechanisms and therapeutic approaches. Semin. Cell Dev. Biol..

[63] Jiang SH, Hu LP, Wang X, Li J, Zhang ZG (2020). Neurotransmitters: emerging targets in cancer. Oncogene.

[64] Grytli HH, Fagerland MW, Fosså SD, Taskén KA (2014). Association between use of β-blockers and prostate cancer-specific survival: a cohort study of 3561 prostate cancer patients with high-risk or metastatic disease. Eur. Urol..

[65] Yang EV, Eubank TD (2013). The impact of adrenergic signaling in skin cancer progression: possible repurposing of β-blockers for treatment of skin cancer. Cancer Biomark..

[66] Phadke S, Clamon G (2019). Beta blockade as adjunctive breast cancer therapy: a review. Crit. Rev. Oncol. Hematol..

[67] Spini A (2019). Evidence of β-blockers drug repurposing for the treatment of triple negative breast cancer: a systematic review. Neoplasma.

[68] Witkiewicz AK (2015). Whole-exome sequencing of pancreatic cancer defines genetic diversity and therapeutic targets. Nat. Commun..

[69] Mauri G (2018). TRKA expression and NTRK1 gene copy number across solid tumours. J. Clin. Pathol..

[70] Cocco E, Scaltriti M, Drilon A (2018). NTRK fusion-positive cancers and TRK inhibitor therapy. Nat. Rev. Clin. Oncol..

[71] Song P (2003). Acetylcholine is synthesized by and acts as an autocrine growth factor for small cell lung carcinoma. Cancer Res..

[72] Song P (2008). Activated cholinergic signaling provides a target in squamous cell lung carcinoma. Cancer Res..

[73] Lin G, Sun L, Wang R, Guo Y, Xie C (2014). Overexpression of muscarinic receptor 3 promotes metastasis and predicts poor prognosis in non-small-cell lung cancer. J. Thorac. Oncol..

[74] Momi N (2013). Nicotine/cigarette smoke promotes metastasis of pancreatic cancer through α7nAChR-mediated MUC4 upregulation. Oncogene.

[75] Choquet D, Triller A (2013). The dynamic synapse. Neuron.

[76] Venkatesh HS (2019). Electrical and synaptic integration of glioma into neural circuits. Nature.

[77] Venkataramani V (2019). Glutamatergic synaptic input to glioma cells drives brain tumour progression. Nature.

[78] Rzeski W, Turski L, Ikonomidou C (2001). Glutamate antagonists limit tumor growth. Proc. Natl Acad. Sci. USA.

[79] Savaskan NE (2008). Small interfering RNA-mediated xCT silencing in gliomas inhibits neurodegeneration and alleviates brain edema. Nat. Med..

[80] Takano T (2001). Glutamate release promotes growth of malignant gliomas. Nat. Med..

[81] Zeng Q (2019). Synaptic proximity enables NMDAR signalling to promote brain metastasis. Nature.

[82] Gambrill AC, Barria A (2011). NMDA receptor subunit composition controls synaptogenesis and synapse stabilization. Proc. Natl Acad. Sci. USA.

[83] Li L, Hanahan D (2013). Hijacking the neuronal NMDAR signaling circuit to promote tumor growth and invasion. Cell.

[84] Kepper M, Keast J (1995). Immunohistochemical properties and spinal connections of pelvic autonomic neurons that innervate the rat prostate gland. Cell Tissue Res..

[85] Folkman J (1971). Tumor angiogenesis: therapeutic implications. N. Engl. J. Med..

[86] De Palma M, Biziato D, Petrova TV (2017). Microenvironmental regulation of tumour angiogenesis. Nat. Rev. Cancer.

[87] Chung HC (2001). Angiogenesis in cancer. Vasc. Health Risk Manag..

[88] Folkman J, Watson K, Ingber D, Hanahan D (1989). Induction of angiogenesis during the transition from hyperplasia to neoplasia. Nature.

[89] Li S (2019). Angiogenesis in pancreatic cancer: current research status and clinical implications. Angiogenesis.

[90] Sharma S, Sharma MC, Sarkar C (2005). Morphology of angiogenesis in human cancer: a conceptual overview, histoprognostic perspective and significance of neoangiogenesis. Histopathology.

[91] Chakroborty D, Sarkar C, Basu B, Dasgupta PS, Basu S (2009). Catecholamines regulate tumor angiogenesis. Cancer Res..

[92] Fiszman GL (2007). Activation of muscarinic cholinergic receptors induces MCF-7 cells proliferation and angiogenesis by stimulating nitric oxide synthase activity. Cancer Biol. Ther..

[93] Chakroborty D (2004). Depleted dopamine in gastric cancer tissues: dopamine treatment retards growth of gastric cancer by inhibiting angiogenesis. Clin. Cancer Res..

[94] Romon R (2010). Nerve growth factor promotes breast cancer angiogenesis by activating multiple pathways. Mol. Cancer.

[95] Lin CY (2014). Brain-derived neurotrophic factor increases vascular endothelial growth factor expression and enhances angiogenesis in human chondrosarcoma cells. Biochem. Pharmacol..

[96] Eichmann A, Brunet I (2014). Arterial innervation in development and disease. Sci. Transl. Med..

[97] Wrona D (2006). Neural-immune interactions: an integrative view of the bidirectional relationship between the brain and immune systems. J. Neuroimmunol..

[98] Rosas-Ballina M (2011). Acetylcholine-synthesizing T cells relay neural signals in a vagus nerve circuit. Science.

[99] Wang H (2003). Nicotinic acetylcholine receptor alpha7 subunit is an essential regulator of inflammation. Nature.

[100] Friedman JR (2019). Acetylcholine signaling system in progression of lung cancers. Pharmacol. Ther..

[101] Cox MA (2019). Choline acetyltransferase-expressing T cells are required to control chronic viral infection. Science.

[102] Salmon H, Remark R, Gnjatic S, Merad M (2019). Host tissue determinants of tumour immunity. Nat. Rev. Cancer.

[103] Darvin P, Toor SM, Sasidharan Nair V, Elkord E (2018). Immune checkpoint inhibitors: recent progress and potential biomarkers. Exp. Mol. Med..

[104] Sharma P, Allison JP (2015). The future of immune checkpoint therapy. Science.

[105] Partecke LI (2013). Induction of M2-macrophages by tumour cells and tumour growth promotion by M2-macrophages: a quid pro quo in pancreatic cancer. Pancreatology.

[106] Sanchez LR (2019). The emerging roles of macrophages in cancer metastasis and response to chemotherapy. J. Leukoc. Biol..

[107] Partecke LI (2016). Chronic stress increases experimental pancreatic cancer growth, reduces survival and can be antagonised by beta-adrenergic receptor blockade. Pancreatology.

[108] Sloan EK (2010). The sympathetic nervous system induces a metastatic switch in primary breast cancer. Cancer Res..

[109] He BP (2006). Differential reactions of microglia to brain metastasis of lung cancer. Mol. Med..

[110] Guldner IH (2020). CNS-Native Myeloid Cells Drive Immune Suppression in the Brain Metastatic Niche through Cxcl10. Cell.

[111] Qian BZ (2011). CCL2 recruits inflammatory monocytes to facilitate breast-tumour metastasis. Nature.

[112] Pyonteck SM (2013). CSF-1R inhibition alters macrophage polarization and blocks glioma progression. Nat. Med..

[113] Blazquez R (2018). PI3K: a master regulator of brain metastasis-promoting macrophages/microglia. Glia.

[114] You H, Baluszek S, Kaminska B (2019). Immune microenvironment of brain metastases-are microglia and other brain macrophages little helpers?. Front. Immunol..

[115] Liu D (2019). Neural regulation of drug resistance in cancer treatment. Biochim. Biophys. Acta Rev. Cancer.

[116] Boshuizen J (2020). Reversal of pre-existing NGFR-driven tumor and immune therapy resistance. Nat. Commun..

[117] Eng JW (2015). Housing temperature-induced stress drives therapeutic resistance in murine tumour models through β2-adrenergic receptor activation. Nat. Commun..

[118] Chen H (2017). beta2-AR activation induces chemoresistance by modulating p53 acetylation through upregulating Sirt1 in cervical cancer cells. Cancer Sci..

[119] Kang Y (2016). Adrenergic stimulation of DUSP1 impairs chemotherapy response in ovarian cancer. Clin. Cancer Res..

[120] Figueroa-Magalhães MC, Jelovac D, Connolly R, Wolff AC (2014). Treatment of HER2-positive breast cancer. Breast.

[121] Smyth EC, Nilsson M, Grabsch HI, van Grieken NC, Lordick F (2020). Gastric cancer. Lancet.

[122] Madrid-Paredes A, Cañadas-Garre M, Sánchez-Pozo A, Calleja-Hernández M (2015). Non-HER2 signaling pathways activated in resistance to anti-HER2 therapy in breast cancer. Breast Cancer Res. Treat..

[123] Shi M (2011). The β2-adrenergic receptor and Her2 comprise a positive feedback loop in human breast cancer cells. Breast Cancer Res. Treat..

[124] Liu D (2016). β2-AR signaling controls trastuzumab resistance-dependent pathway. Oncogene.

[125] Nilsson MB (2017). Stress hormones promote EGFR inhibitor resistance in NSCLC: Implications for combinations with beta-blockers. Sci. Transl. Med..

[126] Wu FQ (2016). ADRB2 signaling promotes HCC progression and sorafenib resistance by inhibiting autophagic degradation of HIF1α. J. Hepatol..

[127] Al-Wadei HA, Al-Wadei MH, Schuller HM (2009). Prevention of pancreatic cancer by the beta-blocker propranolol. Anticancer Drugs.

[128] Powe DG (2010). Beta-blocker drug therapy reduces secondary cancer formation in breast cancer and improves cancer specific survival. Oncotarget.

[129] Heitz F (2013). Impact of beta blocker medication in patients with platinum sensitive recurrent ovarian cancer—a combined analysis of 2 prospective multicenter trials by the AGO Study Group, NCIC-CTG and EORTC-GCG. Gynecol. Oncol..

[130] Smith M (2016). Phase III study of cabozantinib in previously treated metastatic castration-resistant prostate cancer: COMET-1. J. Clin. Oncol..

[131] Collins C (2007). Preclinical and clinical studies with the multi-kinase inhibitor CEP-701 as treatment for prostate cancer demonstrate the inadequacy of PSA response as a primary endpoint. Cancer Biol. Ther..

[132] Shabbir M, Stuart R (2010). Lestaurtinib, a multitargeted tyrosine kinase inhibitor: from bench to bedside. Expert Opin. Invest. Drugs.

